# Opposing Functions of Maspin Are Regulated by Its Subcellular Localization in Lung Squamous Cell Carcinoma Cells

**DOI:** 10.3390/cancers16173009

**Published:** 2024-08-29

**Authors:** Takahiro Matsushige, Tomohiko Sakabe, Hirotoshi Mochida, Yoshihisa Umekita

**Affiliations:** Department of Pathology, Faculty of Medicine, Tottori University, Yonago 683-8505, Japan; matsu-takahiro@tottori-u.ac.jp (T.M.); sakabe@tottori-u.ac.jp (T.S.); aeroheart@tottori-u.ac.jp (H.M.)

**Keywords:** subcellular localization, anti-tumorigenic, pro-tumorigenic, cell invasiveness

## Abstract

**Simple Summary:**

Mammary serine protease inhibitor (maspin) is a tumor suppressor protein, and its nuclear localization is essential for its tumor suppressive activity. We previously reported that cytoplasmic-only maspin expression is an independent unfavorable prognostic indicator in patients with lung squamous cell carcinoma (LUSC). Taken together, we hypothesized that maspin has opposing roles in LUSC depending on its subcellular localization. Maspin was re-expressed in both the nucleus and cytoplasm of LK-2 cells, resulting in significantly decreased cell invasion and migration. In contrast, re-expressed maspin in RERF-LC-AI cells was detected only in the cytoplasm (cytMaspin) and significantly promoted cell invasion and migration. Increased cytMaspin expression downregulates the genes relevant to cell adhesion and activates PYK2 and SRC. This study suggests that the cytoplasm-to-nuclear translocation of maspin is dysregulated in RERF-LC-AI cells, and cytMaspin enhances the invasive potential by activating PYK2 and SRC in LUSC.

**Abstract:**

Mammary serine protease inhibitor (maspin) is a tumor suppressor protein downregulated during carcinogenesis and cancer progression; cytoplasmic-only maspin expression is an independent, unfavorable prognostic indicator in patients with lung squamous cell carcinoma (LUSC). We hypothesized that the cytoplasmic-only localization of maspin has tumor-promoting functions in LUSC. The subcellular localization of maspin and the invasive capability of LUSC cell lines were investigated using RNA sequencing (RNA-seq), Western blotting, and siRNA transfection. Maspin mRNA and protein expression were suppressed in LK-2 and RERF-LC-AI cells. Cell invasion significantly increased in response to siRNA-mediated maspin knockdown in KNS-62 cells expressing both nuclear and cytoplasmic maspin. In LK-2 cells, both nuclear and cytoplasmic maspin were re-expressed, and cell invasion and migration were significantly decreased. In contrast, re-expressed maspin in RERF-LC-AI cells was detected only in the cytoplasm (cytMaspin), and cell invasion and migration were significantly promoted. RNA-seq and downstream analyses revealed that increased cytMaspin expression downregulated the genes associated with cell adhesion and activated PYK2 and SRC, which play important roles in cancer progression. Our study demonstrates a novel biological function of cytMaspin in enhancing the invasive capabilities of LUSC cells. Understanding cytoplasm-to-nuclear maspin translocation dysregulation may develop novel therapeutic approaches to improve the prognosis of patients with LUSC.

## 1. Introduction

Lung cancer is the leading cause of cancer morbidity and mortality worldwide with GLOBOCAN estimating that approximately 2,4 million new cases of lung cancer will be diagnosed and almost 1.8 million will die by 2022 globally [1]. Non-small cell lung cancer (NSCLC) accounts for most primary lung cancers (approximately 80–85%), of which 20–30% are squamous cell carcinomas (SCCs) [2]. Although *FGFR*, *DDR2*, *PTEN*, and *PIK3CA* gene aberrations are frequently observed in lung SCCs (LUSCs) [3,4], common driver mutations, such as *EGFR* observed in lung adenocarcinoma (LUAD), have rarely been identified in LUSCs, and no approved effective molecular targeted therapies are available currently [5]. Alternatively, with the recent development of programmed death-1 (PD-1)/programmed death-ligand 1 (PD-L1) inhibitors, immunotherapy for NSCLC has demonstrated efficacy in improving the long-term prognosis of patients [6]. However, even with combined immunotherapy and chemotherapy, the median overall survival of patients with metastatic LUSC is 17.1 months [7]. Therefore, the therapeutic efficacy of current treatments for LUSC remains unsatisfactory, and a detailed elucidation of the molecular events involved in LUSC carcinogenesis and progression is necessary to develop effective therapies.

Mammary serine protease inhibitor (maspin) was originally identified as a protein encoded by the *SERPINB5* gene located on the human chromosome 18q21.3 [8]. Maspin is a member of the serine protease inhibitor (serpin) superfamily due to its similarity in protein structure, containing the reactive center loop (RCL) required to recognize proteases and inhibit their activity; however, the RCL of maspin is shorter than that of other serpins and thus cannot inhibit protease activity. Maspin expression markedly decreases during carcinogenesis and progression in some cancers [9,10]. Accumulating evidence has shown that maspin is a downstream target of p53 and PTEN, which are well-known tumor suppressor genes [11,12]. Increased maspin expression in cancer cells reportedly induces cell cycle arrest and apoptosis, promotes drug sensitivity, and inhibits HDAC1 activity, which play crucial roles in cancer progression and development [13,14]. Furthermore, the inhibition of HDAC1 by maspin reportedly promotes p53 acetylation and increases its transcriptional activity [15]. Therefore, *SERPINB5* is considered a tumor suppressor gene. We have focused on the correlation between the subcellular localization of maspin expression and clinicopathological variables, especially patient prognoses in several cancer types, including LUSC, LUAD, breast cancer, pancreatic cancer, and oral SCC [16,17,18]. In LUSC, as in other cancers, we demonstrated that cytoplasmic-only (cytMaspin), but not combined nuclear and cytoplasmic (panMaspin), maspin expression is an independent unfavorable prognostic indicator in patients with LUSC [16]. In addition, cytMaspin plays an important role in promoting the invasion and induces epithelial–mesenchymal transition (EMT) through SRGN/TGFβ2 signaling axis activation in the MDA-MB-231 human breast carcinoma cell line [19]. Goulet et al. reported that nuclear maspin localization in highly metastatic human breast carcinoma cell lines was indispensable for its tumor-suppressive activity [20]. Taken together, we hypothesized that maspin plays opposing roles, namely, in the inhibitory function of panMaspin and the promoting function of cytMaspin, in SCC progression. To our knowledge, no prior reports have focused on whether the opposing function of maspin is dependent on its subcellular localization in LUSC cells; therefore, we aimed to elucidate the subcellular localization-dependent role of maspin.

## 2. Materials and Methods

### 2.1. Cell Culture

Human LUSC cell lines were obtained from the RIKEN BioResource Research Center (Ibaraki, Japan) (LC-1/sq, LK-2, and RERF-LC-AI) and the Japanese Collection of Research Bioresources Cell Bank (Osaka, Japan) (EBC-1, HARA, and KNS-62), respectively. LK-2, KNS-62, EBC-1, and HARA cells were maintained in Roswell Park Memorial Institute 1640 (RPMI1640; Thermo Fisher Scientific, Waltham, MA, USA). RERF-LC-AI cells were cultured in Eagle’s minimum essential medium (Sigma-Aldrich, St. Louis, MO, USA). RPMI1640/Ham’s F12 (Thermo Fisher Scientific), mixed (1:1) medium containing 25 mM HEPES (Thermo Fisher Scientific), was used to culture LC-1/sq cells. 293FT cells were purchased from Thermo Fisher Scientific and maintained in Dulbecco’s modified Eagle’s medium (DMEM; Nissui Pharmaceutical, Tokyo, Japan). All media were supplemented with 10% inactivated fetal bovine serum (FBS; Biological Industries, Cromwell, CT, USA). All cell lines were cultured under standard conditions: 5% CO_2_ at 37 °C. The authenticity of the cell lines was verified using the Cross-Contaminated or Misidentified Cell Lines Database provided by the International Cell Line Authentication Committee [21] to confirm that the cell lines used in this study were not on the list.

### 2.2. Real-Time Quantitative Polymerase Chain Reaction (RT-qPCR)

Total RNA was isolated from LUSC cell lines grown in six-well plates by using TRIzol Reagent (Thermo Fisher Scientific). The conversion from RNA to cDNA was accomplished utilizing a High-Capacity RNA-to-cDNA kit (Thermo Fisher Scientific). Levels of mRNA expression were measured on a LightCycler 96 system (Roche Diagnostics, Mannheim, Germany) by using TaqMan Gene Expression Master Mix (Thermo Fisher Scientific) according to the manufacturer’s instructions. The primer–probe information used in this study is detailed in Supplementary Table S1. *GAPDH* expression levels was used as an internal reference for normalization.

### 2.3. Western Blotting

Radioimmunoprecipitation assay (RIPA) buffer with a protease inhibitor (Roche Diagnostics) and a phosphatase inhibitor cocktail (Roche Diagnostics) was used to extract the total protein from whole cells. For the subcellular protein fraction assay, the nuclear and cytoplasmic fractions were extracted with the cytoplasmic extraction buffer (CEB) and nuclear extraction buffer (NEB) provided with the Subcellular Protein Fractionation Kit for Cultured Cells (Thermo Fisher Scientific). Following the manufacturer’s instructions, soluble cytoplasmic proteins were extracted by increasing the permeability of the cell membrane with ice-cold CEB buffer added to the cell pellet; then, membrane extraction buffer was added to dissolve plasma, mitochondria, and ER–Golgi membranes other than nuclear membranes, and finally, the soluble nuclear proteins were extracted by dissolving nuclei recovered by centrifugation in NEB buffer. Samples were prepared in 5× sample buffer and RIPA buffer after the protein concentration was determined by Bradford protein assay (Bio-Rad Laboratories, Hercules, CA, USA). Protein samples boiled for 5 min were separated by sodium dodecyl sulfate polyacrylamide gel electrophoresis, and then transferred to 0.45 μm polyvinylidene fluoride (PVDF) membranes (Merck Millipore, Darmstadt, Germany). Before blocking with ECL Prime Blocking Reagent (GE Healthcare, Buckinghamshire, UK), PVDF membranes were cut and then incubated overnight at 4 °C with the primary antibodies. The primary antibodies used in this study are listed in Supplementary Table S2. The PVDF membrane was incubated with peroxidase-linked secondary antibodies for 1 h at room temperature (RT), and protein bands were detected using ECL Prime Western Blotting Detection Reagent (GE Healthcare).

### 2.4. Immunofluorescence

LUSC cells were plated in Nunc Lab-Tek II eight-well chamber slides (Thermo Fisher Scientific) and incubated overnight at 37 °C and 5% CO_2_ to facilitate attachment. Cells were fixed with 4% paraformaldehyde/phosphate-buffer saline (PBS) for 15 min. Samples were permeabilized by treatment with 100% ice-cold methanol at −20 °C for 10 min followed by 0.2% Triton-X/PBS at RT for 10 min. After blocking by treatment with 10% goat serum/PBS at RT for 60 min, samples were immunolabeled with anti-maspin antibody diluted in PBS at 4 °C overnight; then, they were immunolabeled with Alexa Fluor 488- or Alexa Fluor 647-conjugated secondary antibody (both from Thermo Fisher Scientific) at RT for 60 min. The samples were coverslipped with a ProLong Diamond Antifade Mountant containing DAPI (Thermo Fisher Scientific). Confocal fluorescent images were obtained using a Zeiss LSM780 confocal microscope (Carl Zeiss, Baden-Württemberg, Germany).

### 2.5. siRNA Transfection

KNS-62 cells (3.6 × 10^5^ cells/well) were seeded in six-well plates and incubated overnight at 37 °C and 5% CO_2_ to facilitate attachment. Control siRNA (Silencer Negative Control #1, Thermo Fisher Scientific) or maspin siRNA (Silencer Select siRNA, s10466 and s10468, Thermo Fisher Sicentific) was transiently transfected using Lipofectamine RNAiMAX (Thermo Fisher Scientific) and Opti-MEM I Reduced Serum Medium (Thermo Fisher Scientific) to a final concentration of 50 nM according to the manufacturer’s instructions. Protein extraction for Western blotting and transwell invasion assay were then performed 48 and 24 h after siRNA transfection, respectively.

### 2.6. Establishment of RERF-LC-AI and LK-2 Cell Lines with High Expression of Maspin

The construction of lentivirus-based expression plasmids (pLenti/ZsGreen and pLenti/maspin-ZsGreen) and the production of lentiviral vectors has been previously described [19]. LK-2 and RERF-LC-AI cells were transfected with the lentivirus-based vectors at 25 multiplicity of infection. To select cells with resistance, blasticidin (Fujifilm Wako Pure Chemical Corporation, Osaka, Japan) was applied at a final concentration of 10 μg/mL to select cells with resistance. LK-2-control (LK2-ctrl), LK-2-maspin (LK2-maspin), RERF-LC-AI-control (LC-AI-ctrl), and RERF-LC-AI-maspin (LC-AI-maspin) were established by pooling surviving clones.

### 2.7. Cell Invasion Assay

KNS-62 cells transfected with siRNA or established stable LUSC cell lines were serum starved for 24 h in serum-free medium. Cells with serum-free medium were placed in the upper chamber of a transwell insert with an 8 μm pore size membrane in a 24-well plate. The culture medium containing 10% inactivated FBS was added to the lower chambers. After 24 (LC-AI-ctrl and LC-AI-maspin) or 72 (others) h incubation, non-migrating cells were removed from the upper chamber with a cotton swab. The invaded cells were stained with crystal violet included in the QCM ECMatrix Cell Invasion Assay, 24-well (8 μm), colorimetric kit (Merck Millipore, Billerica, MA, USA) according to the manufacturer’s instructions. Whole membrane images were captured using a BZ-X800 all-in-one fluorescence microscope (Keyence, Osaka, Japan). The invading cells were counted using the ImageJ/Fiji software (ver. 2.1.0/1.53c).

### 2.8. Cell Migration Assay

Established stable cell lines treated with 1 mg/mL mitomycin C for 2 h to inhibit cell proliferation were seeded into culture inserts in two-well plates in a μ-Dish 35 mm High (ibid GmbH, Gräfelfing, Germany) and incubated overnight at 37 °C and 5% CO_2_ to facilitate attachment. After cell attachment, the culture inserts were removed with sterile tweezers, and the dish was filled with culture medium followed by incubation. Images at 0 and 12 (LC-AI-ctrl and LC-AI-maspin) or 24 h (LK-2-ctrl and LK-2-maspin) after incubation were acquired with an IX73 inverted microscope (Olympus Corporation, Tokyo, Japan). The wound area was quantified in the Wound_healing_size_tool plugin [22] for ImageJ/Fiji software and compared with the initial gap area at 0 h using the following formula: wound closure (%) = [(wound area at time 0) − (wound area at time x)/(wound area at time 0)] × 100.

### 2.9. Transcriptome Analysis (mRNA-Seq)

The total RNA from LK2-ctrl, LK-maspin, LC-AI-control, and LC-AI-maspin cells was recovered using the RNeasy Mini Kit (QIAGEN, Valencia, CA, USA) according to the manufacturer’s instructions. Isolated RNA was sent to Eurofins Genomics (Tokyo, Japan) for RNA-Seq analysis. RNA was fragmented after poly (A) purification and reverse-transcribed into cDNA using random primers. The synthesized cDNA was prepared as a standard-specific mRNA library after adapter addition and fragmentation, and mRNA-seq analysis was performed using a NovaSeq 6000 system (Illumina, San Diego, CA, USA). Sequence reads were cleaned using the Trimmomatic software (ver. 0.39) and mapped to a reference sequence (GRCh38.p13) using BWA (ver. 0.7.17). Using edgeR (ver.3.16.5), the data were normalized using the Trimmed Mean of M-values method, and statistical analysis with a likelihood ratio test was performed. Based on the statistical analysis, an FDR-adjusted *p* < 0.05 was used to estimate statistical significance. Genes with log_2_(fold change) >0 and <0 were set as upregulated and downregulated genes, respectively, in LC-AI-maspin. The mRNA-seq datasets provided by Eurofins have been deposited in the NCBI for Biotechnology Information Gene Expression Omnibus (GEO) repository (GSE180712). KEGG (Kyoto Encyclopedia of Genes and Genomes) pathway enrichment analysis was performed to interpret the biological functions of the differentially expressed genes (DEGs) using an online Database for Annotation, Visualization, and Integrated Discovery (https://david.ncifcrf.gov/home.jsp, accessed on 10 January 2023). Protein–protein interaction (PPI) networks constructed using Search Tool for the Retrieval of Interacting Genes/Proteins (STRING; http://www.string-db.org/, accessed on 31 January 2023) were visualized using Cytoscape 3.9.1.

### 2.10. Statistical Analysis

Data are presented as mean ± standard deviation. Statistical differences between the means of each group were evaluated by Student’s *t*-test or Dunnett’s test; *p* < 0.05 was considered statistically significant.

## 3. Results

### 3.1. Expression Status and Subcellular Localization of Maspin in LUSC Cell Lines

**Figure 1:**
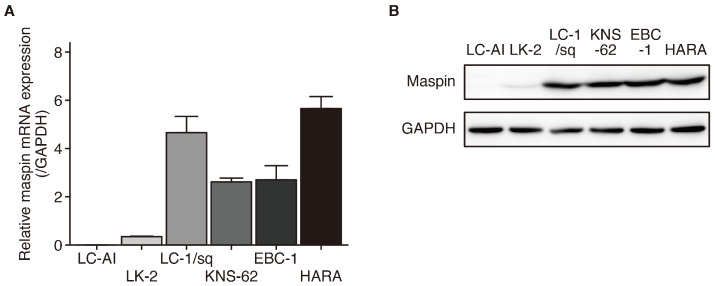

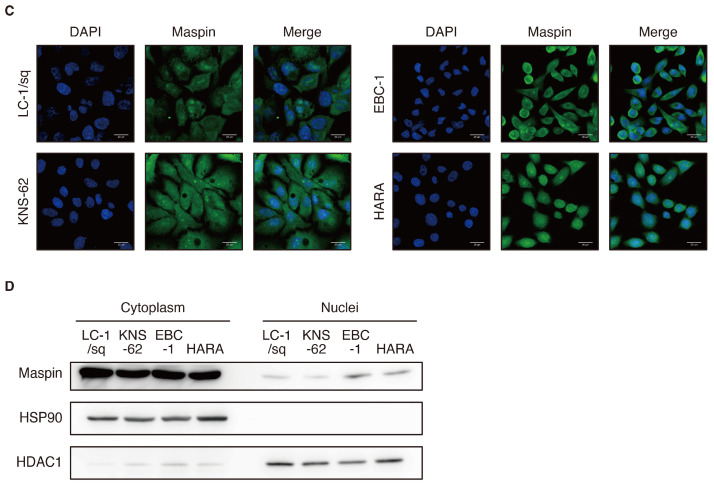
Maspin expression in LUSC cell lines. (**A**) Maspin mRNA expression levels in six LUSC cell lines (*n* = 3). Data were normalized to level of *GAPDH*. (**B**) Maspin and GAPDH (loading control) protein expression levels in whole-cell lysates of LUSC cell lines. Whole Western blot images are presented in Supplementary Figure S1. (**C**) Representative images from immunofluorescence staining of maspin (green) and nuclei (blue) in LUSC cell lines. Scale bar: 20 μm. (**D**) Maspin, HSP90 (loading control for cytoplasm), and HDC1 (loading control for nuclei) protein expression levels in cytoplasmic and nuclear lysates of LUSC cell lines. Whole Western blot images are presented in Supplementary Figure S1. LUSC, lung squamous cell carcinoma; Maspin, mammary serine protease inhibitor.

To clarify maspin expression in LUSC, mRNA and protein expression were investigated in six different LUSC cell lines. Of the six cell lines, maspin mRNA expression was suppressed in LK-2 and RERF-LC-AI cell lines, and slightly detected in LK-2 cells, but not in RERF-LC-AI cells (Figure 1A). In contrast, LC-1/sq, KNS-62, EBC-1, and HARA cell lines exhibited high maspin expression. In these cell lines, maspin protein expression exhibited a profile similar to that of its corresponding mRNA expression (Figure 1B). Next, we investigated the subcellular localization of the maspin protein in LUSC cell lines expressing maspin. Immunofluorescence (IF) staining showed that maspin was expressed in both the nucleus and cytoplasm (panMaspin) of all four LUSC cell lines (Figure 1C). Consistent with these results, Western blotting of protein samples fractionated into the nucleus and cytoplasm showed similar subcellular localizations (Figure 1D). These results suggest that maspin expression is suppressed in some LUSC cell lines, but it is expressed in both the nucleus and cytoplasm of maspin-expressing cell lines similar to the subcellular localization observed in the normal human bronchial epithelial cell line [23].

### 3.2. Promotion of Invasive Capability by panMaspin Knockdown in LUSC Cell Line

**Figure 2:**
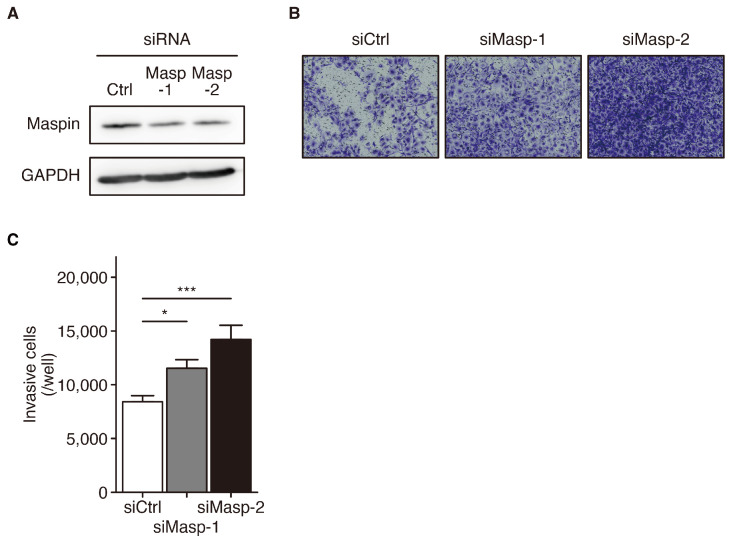
Promotion of the cell invasion capability by pancellular maspin knockdown in KNS-62 cells. (**A**) Maspin and GAPDH (loading control) protein expression levels in whole-cell lysates of KNS-62 cells transfected with siRNA targeting negative control (Ctrl), maspin-1 (Masp-1), and maspin-2 (Masp-2) for 24 h. Whole Western blot images are presented in Supplementary Figure S2. (**B**) Representative images of a transwell invasion assay performed using KNS-62 cells with negative control siRNA (siCtrl), maspin-1 siRNA (siMasp-1), and maspin-2 siRNA (siMasp-2). (**C**) The bar graph represents the average number of invading KNS-62 cells per well. * *p* < 0.05, *** *p* < 0.001 vs. siCtrl; Dunnett’s test (*n* = 3). Maspin, mammary serine protease inhibitor.

Nuclear maspin expression reportedly exhibits tumor-suppressive capabilities [20]. Therefore, we examined the alterations in cell invasion induced by panMaspin knockdown to determine its role in the LUSC phenotype. In KNS-62 cells expressing panMaspin, the maspin protein was knocked down using two siRNAs targeting maspin (Figure 2A). Cell invasion significantly increased in response to maspin knockdown in KNS-62 cells (Figure 2B,C), suggesting that maspin expressed in both the nucleus and cytoplasm is involved in LUSC suppression and cell invasion inhibition.

### 3.3. Maspin Has Subcellular Localization-Dependent Opposing Effects on Cell Invasion and Migration

**Figure 3:**
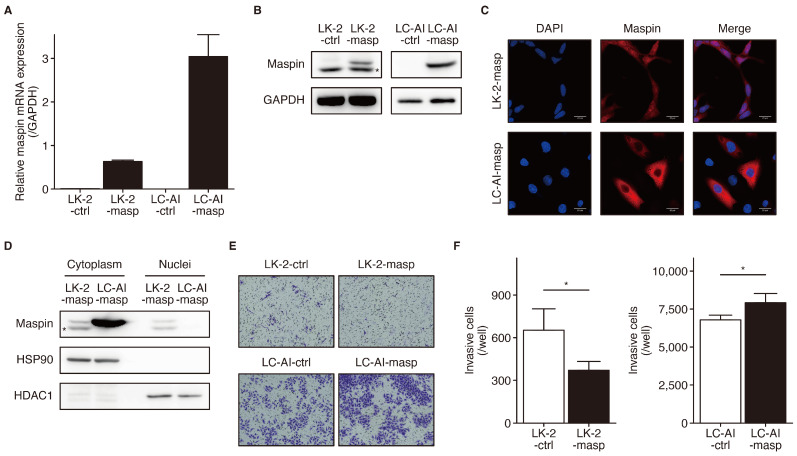

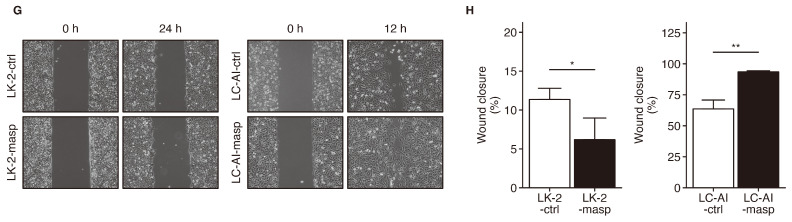
Subcellular localization-dependent opposing roles of maspin on cell invasion and migration capability. (**A**) *Maspin* mRNA expression levels in LK-2 and RERF-LC-AI cells stably transfected with ZsGreen (-ctrl) or maspin-ZsGreen (-masp). (*n* = 3). Data were normalized to level of *GAPDH*. (**B**) Maspin and GAPDH (loading control) protein expression levels in whole-cell lysates of LK-2 and RERF-LC-AI cells stably transfected with ZsGreen (-ctrl) or maspin-ZsGreen (-masp). Whole Western blot images are presented in Supplementary Figure S3. The asterisk marks a non-specific band present only in the LK-2 cell line. (**C**) Representative images from immunofluorescence staining of maspin (green) and nuclei (blue) in LK2-masp and LC-AI-masp cells. (**D**) Maspin, HSP90 (loading control for cytoplasm), and HDC1 (loading control for nuclei) protein expression levels in cytoplasmic and nuclear lysates of LK2-masp and LI-AI-masp cells. Whole Western blot images are presented in Supplementary Figure S3. The asterisk marks a non-specific band present only in LK-2 cell line. (**E**) Representative images of a transwell invasion assay performed using LK-2 and RERF-LC-AI cells stably transfected with ZsGreen (-ctrl) or maspin-ZsGreen (-masp). (**F**) The bar graph represents the average number of invading LK-2 (left panel) and RERF-LC-AI (right panel) cells stably transfected with ZsGreen (-ctrl) or maspin-ZsGreen (-masp) per well. * *p* < 0.05 vs. ctrl; Students’ *t*-test (*n* = 3). Maspin, mammary serine protease inhibitor. (**G**) Representative images of a wound-healing assay performed using LK-2 and RERF-LC-AI cells stably transfected with ZsGreen (-ctrl) or maspin-ZsGreen (-masp). (**H**) The bar graph represents the percentage of wound closure after 24 and 12 h for LK-2 (left panel) and RERF-LC-AI (right panel) cells stably transfected with ZsGreen (-ctrl) or maspin-ZsGreen (-masp), respectively. * *p* < 0.05, ** *p* < 0.01 vs. ctrl; Students’ *t*-test (*n* = 3). Maspin, mammary serine protease inhibitor.

To investigate how maspin re-expression affects cell invasiveness, LK-2 and RERF-LC-AI cells, in which endogenous maspin expression was suppressed, were used to establish stable cell lines for maspin. In the established cell lines (LK2-maspin and LC-AI-maspin), maspin mRNA and protein expression was markedly higher than in the control cell lines (Figure 3A,B). The examination of subcellular localization by IF staining in LK2-maspin cells revealed that maspin was present in both the nucleus and cytoplasm as well as endogenous maspin expression in KNS-62 cells (Figure 3C). Notably, maspin was mainly detected in the cytoplasm, but not nucleus, of LC-AI-maspin cells (Figure 3C). These subcellular localizations were also similar in Western blot analysis of the nuclear and cytoplasmic fractions, and expression in the nuclear fraction was observed in LK2-maspin cells but not in LC-AI-maspin cells (Figure 3D). Therefore, the nuclear translocation process of maspin is inhibited in LC-AI-maspin cells by the disruption of the regulatory mechanism involved in subcellular localization. The invasion ability of LK2-maspin cells stably expressing panMaspin was significantly decreased compared to that of the control. In contrast, increased cytMaspin expression in LC-AI-maspin cells promoted cell invasion (Figure 3E,F). Consistent with these results, the overexpression of panMaspin and cytMaspin showed similar results in the investigation of cell migration ability using wound-healing assay (Figure 3G,H). These results suggest that the role of maspin in LUSC is dependent on its subcellular localization, and it is generally expressed in both the nucleus and cytoplasm to suppress cell invasion and migration; however, its loss of expression in the nucleus promotes cell invasion and migration.

### 3.4. Maspin Re-Expression-Induced Alterations in Gene Expression in LUSC Cell Lines

**Figure 4:**
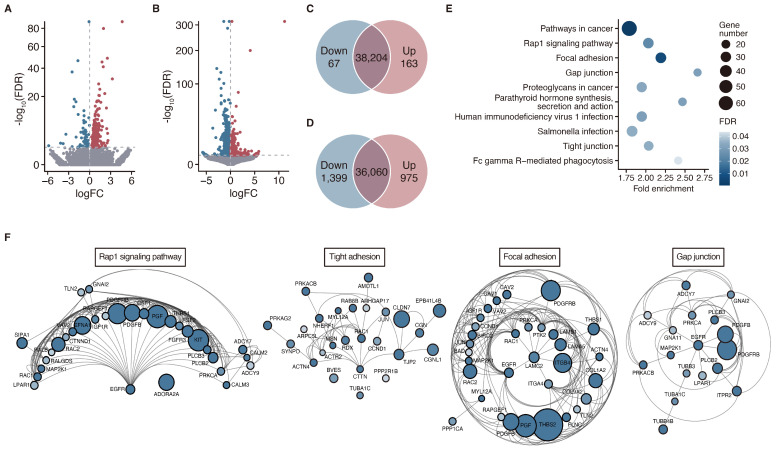
Transcriptome analysis in LUSC cells stably transfected with ZsGreen or maspin-ZsGreen. (**A**,**B**) Volcano plot for DEGs representing distribution of fold change [*x*-axis; log_2_(fold change)] and significance [*y*-axis; −log_10_(FDR)]. Dots show the upregulated (red), downregulated (blue), and unchanged (gray) DEGs in LK2-maspin (**A**) and LC-AI-maspin cells (**B**). (**C**,**D**) Venn diagram showing the number of DEGs in LK2-maspin (**C**) and LC-AI-maspin cells (**D**). (**E**) Bubble chart for pathway analysis based on the DAVID KEGG pathway, showing top-10 pathway enriched in a list of downregulated DEGs in LC-AI-maspin cells. The *x*-axis represents the fold enrichment values, while the *y*-axis represents the enrichment term. Bubble size and transparency represent the number of DEGs and FDR adjusted *p*-values, respectively. (**F**) Visualization of STRING protein–protein networks with downregulated DEGs of LC-AI-maspin assigned to four signaling pathways involved in cell adhesion. Size and color depth of the dots were proportional to the fold change and FDR-adjusted *p*-values. DAVID, Database for Annotation, Visualization, and Integrated Discovery; DEGs, differentially expressed genes; FDR, false discovery rate; KEGG, Kyoto Encyclopedia of Genes and Genomes; LUSC, lung squamous cell carcinoma.

To reveal the association between maspin re-expression and LUSC cell invasion and migration regulation, transcriptome analysis was performed using established stable cell lines. To identify the genes whose expression profiles were altered by maspin overexpression, a likelihood ratio test was performed to identify differentially expressed genes (DEGs) in both LK-2 and RERF-LC-AI cells. In total, 230 (Figure 4A) and 2374 (Figure 4B) genes were identified as significant DEGs (false discovery rate [FDR] adjusted *p* < 0.05) in LK-2 and RERF-LC-AI cells, respectively. In panMaspin-overexpressing LK-2 cells, 163 and 67 genes were significantly upregulated and downregulated, respectively (Figure 4C, Supplementary Table S3). Simultaneously, the expression of 975 and 1399 genes significantly increased or decreased, respectively, in LC-AI-maspin cells overexpressing cytMaspin (Figure 4D, Supplementary Table S4). To gain further insight into the biological interpretation of the DEGs in LK2-maspin and LC-AI-maspin cells, KEGG pathway analysis was performed. Analysis with FDR adjusted *p* < 0.05 as a threshold indicated that upregulated DEGs in LK2-maspin were significantly associated with “Estrogen signaling pathway (hsa04915)”, while downregulated DEGs showed no association with any pathway (Supplementary Table S5). In addition, upregulated and downregulated DEGs in the LC-AI-maspin cells were significantly enriched in 1 and 11 signaling pathways, respectively (Supplementary Table S6). Notably, pathway analysis revealed that among the downregulated DEGs in LC-AI-maspin cells, 71 were mainly associated with signaling pathways involved in cell adhesion, such as the Rap1 signaling pathway, focal adhesion, tight junctions, and gap junctions (Figure 4E). To explore the interactions of the genes assigned to the cell adhesion signaling pathways, a PPI network was constructed using the STRING database. Although some genes overlapped in the four signaling pathways, the PPI networks of Rap1 signaling, focal adhesion, tight junction, and gap junction pathways were identified (Figure 4F), suggesting that the suppression of the expression of genes involved in cell adhesion by increased cytMaspin expression is associated with the enhanced cell invasive capability of LC-AI-maspin.

### 3.5. PYK2 and SRC Activation in LC-AI-Maspin Cells

**Figure 5:**
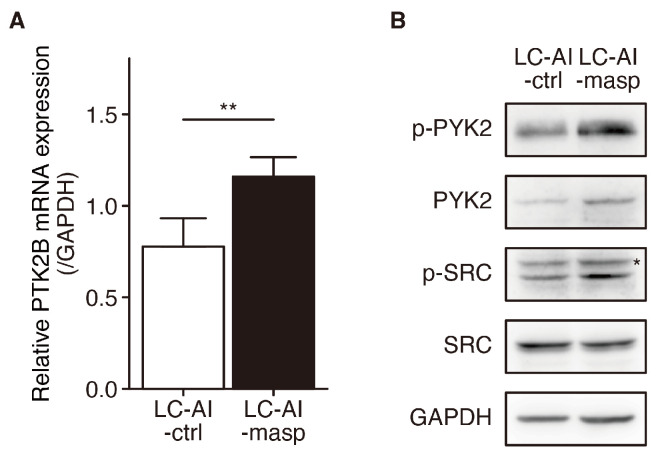
PYK2 and SRC expression and phosphorylation levels in RERF-LC-AI cells with or without maspin overexpression. (**A**) *PTK2B* mRNA expression in RERF-LC-AI cells stably transfected with ZsGreen or maspin-ZsGreen. Data were normalized to *GAPDH* level. ** *p* < 0.01 vs. ctrl; Student’s *t*-test (*n* = 4). (**B**) PYK2, SRC, and GAPDH protein expression and phosphorylation (loading control) in whole-cell lysates of RERF-LC-AI cells stably transfected with ZsGreen (-ctrl) or maspin-ZsGreen (-masp). Whole Western blot images are presented in Supplementary Figure S4. The asterisk marks a non-specific band present in both cell lines.

To gain further insight into the molecular mechanisms underlying the enhanced cell-invasive capability of LC-AI-maspin, the phosphorylation and activation of proteins whose expression was upregulated in LC-AI-maspin were investigated. The expression of *PTK2B*, which was upregulated in LC-AI-maspin cells in the NGS analysis, was examined using qRT-PCR. *PTK2B* mRNA expression levels in LC-AI-maspin cells were significantly higher than that in the LC-AI-control cells (Figure 5A). *PTK2B* encodes the PYK2 protein, a member of the non-receptor tyrosine kinase of the focal adhesion kinase (FAK) family, which reportedly plays a pivotal role in carcinogenesis and cancer progression [24]. Therefore, the PYK2 protein phosphorylation level was examined in LC-AI-control and LC-AI-maspin cells. PYK2 phosphorylation at Tyr402, a reliable PYK2 activation indicator, consistently increased in LC-AI-maspin cells (Figure 5B). PYK2 activation is initiated by Tyr402 phosphorylation, which is followed by binding to the SRC homology 2 (SH2) domain of the SRC family [25]. Therefore, we examined SRC activation in LC-AI-control and LC-AI-maspin cells using Western blotting. The Tyr402 phosphorylation level in LC-AI-maspin cells was significantly higher than that in the LC-AI-control cells (Figure 5B). These results suggest that increased cytMaspin expression promotes RERF-LC-AI cell invasion and migration via PYK2 and SRC activation.

## 4. Discussion

The largest series, including 209 patients with LUSC, showed that cytoplasmic or nuclear maspin expression did not correlate with tumor-specific survival [26]. In turn, enhanced cytoplasmic maspin expression is a significantly favorable prognostic factor [27], whereas cytoplasmic and nuclear staining are independent favorable prognostic indicators [28]. We have previously reported that cytoplasmic-only maspin expression predicts an unfavorable prognosis in patients with LUSC [16]. Thus, whether cytoplasmic localization of maspin contributes to SCC progression remains unknown.

In KNS-62 cells, which highly express maspin in both the nucleus and cytoplasm, maspin knockdown enhanced cell invasion. Furthermore, panMaspin re-expression in LK2-maspin cells significantly suppressed cell invasiveness. These findings agree with that of a previous report that nuclear maspin acts as a tumor suppressor [20]. In contrast, cytMaspin re-expression in LC-AI-maspin cells significantly enhanced cell invasiveness. These results agree with our previous report that cytoplasmic-only maspin expression predicts unfavorable prognosis in patients with LUSC [16].

Maspin undergoes various post-translational modifications (PTMs), including S-nitrosylation [29], acetylation [30], and phosphorylation [31,32]. Although the molecular mechanisms involved in regulating subcellular maspin localization remain controversial, accumulating evidence suggests that PTMs of maspin regulate its subcellular localization. Longhi et al. [33,34] showed that activation of the PI3K–AKT and JAK2–STAT3 pathways, but not the MAPK pathway, by enhancing the EGFR signaling pathway induced maspin phosphorylation, increasing nuclear maspin accumulation in a non-tumorigenic human mammary epithelial cell line (MCF10A). We observed that overexpressed maspin was expressed in both the nucleus and cytoplasm in LK-2 cells, whereas it was expressed only in the cytoplasm, but not in the nucleus, in RERF-LC-AI cells. It is well known that RERF-LC-AI cells harbor EGFR driver mutation, while LK-2 cells are wild type, suggesting that alterations in downstream signaling pathways due to EGFR driver mutation may also affect the regulation of subcellular localization of maspin. Notably, LC-AI-maspin cells overexpressing cytMaspin showed an increased expression of genes involved in the MAPK pathway and decreased expression of genes involved in the PI3K–AKT and mTOR pathways, including EGFR. These results suggest that the maspin phosphorylation profile is involved in regulating its subcellular localization in LUSC cells, which is similar to that in normal cells. Stimulation by cell–cell contact also predominates over the signaling pathway activation by EGF in regulating nuclear maspin accumulation in human non-transformed cell lines [34]. In contrast, LC-AI-maspin cells exhibited a decreased expression of genes involved in cell adhesion. In tumor cells, the dysregulation of various intracellular signaling pathways regulates cellular functions through activating signaling cascades that are completely different from those in normal cells [35,36,37]. These findings may also be explained by differences in the intracellular signaling pathways between normal and cancer cells. Further studies on cancer and normal cells should elucidate the regulatory mechanisms underlying the subcellular maspin localization.

Although several molecular mechanisms underlying tumor suppression by nuclear maspin have been reported, those involved in tumor promotion by cytoplasmic maspin remain largely unknown. We demonstrated that in LC-AI-maspin cells, along with increased cytMaspin expression, PYK2 and SRC activation was enhanced due to increased phosphorylation. PYK2 is aberrantly expressed in many malignant tumors, including lung cancer, compared to that in normal tissues, and it plays a critical role in regulating cell proliferation, migration, invasion, and adhesion [24]. During cancer progression, PYK2 acts as a hub downstream of various signaling cascades mediated by receptor tyrosine kinases, integrins, cytokines, and chemokines. PYK2 is essential for promoting the expression of matrix metalloproteinases, which play an essential role in degrading the extracellular matrix (ECM) during these signaling cascades [38]. Moreover, focal adhesion formation is essential for cell–ECM and cell–cell adhesion, and their turnover mediates cell migration [39,40]. Hashido et al. [41] demonstrated that the intracellular Ca^2+^ concentration induces PYK2 accumulation during focal adhesion, resulting in focal adhesion disassembly, thereby inhibiting cell–cell adhesion formation and promoting cell–cell repulsion, which in turn promotes cell motility. The activation of PYK2 and SRC is known to attenuate epithelial cell assembly, maintenance, and cadherin-based cell–cell adhesion [42,43]. Our observation that gene expression involved in cell adhesion was downregulated in LC-AI-maspin, which is consistent with these previous findings suggesting that the activation of intracellular signaling cascades centered on PYK2 by cytMaspin is involved in LUSC progression. Recently, Liu et al. showed that maspin overexpression in colorectal cancer induces EMT via the TNF-α/NF-κB signaling pathway [44]. Although further investigations are needed to understand the detailed molecular mechanisms by which cytMaspin increases PYK2 expression and promotes its activation, our findings and the fact that EMT increases PYK2 expression [45] suggest that EMT may be involved in the activation of PYK2 and SRC by cytMaspin in LUSC cells.

## 5. Conclusions

We demonstrated that panMaspin inhibits the invasive potential of the human LUSC cell line, whereas cytMaspin plays an important role in enhancing the invasive potential by downregulating the genes associated with cell adhesion and activating PYK2 and SRC. Our findings suggest that understanding the regulatory mechanisms of the cytoplasm-to-nuclear translocation of maspin and the dysregulation of this mechanism in cancer may provide new insights into the controversial role of maspin in cancer cells.

## Data Availability

RNA-seq data are available in the GEO database under accession number GSE180712. The data used in this manuscript are available from the corresponding author on email request.

## Supplementary Materials

The following supporting information can be downloaded at https://www.mdpi.com/article/10.3390/cancers16173009/s1, Figure S1: Whole Western blot images in Figure 1; Figure S2: Whole Western blot images in Figure 2; Figure S3: Whole Western blot images in Figure 3; Figure S4: Whole Western blot images in Figure 5; Table S1: List of primer and probe sets used in RT-qPCR; Table S2: List of primary antibodies used in Western blot; Table S3: List of 230 significantly differentially expressed genes in LK2-maspin; Table S4: List of 2374 significantly differentially expressed genes in LC-AI-maspin; Table S5: List of KEGG pathways assigned to DEGs in LK2-maspin; Table S6: List of KEGG pathways assigned to DEGs in LC-AI-maspin.
