# Supplementary material for: Opposing Functions of Maspin Are Regulated by Its Subcellular Localization in Lung Squamous Cell Carcinoma Cells
Source: Cancers (Basel). 2024 Aug 29;16(17):3009. doi: 10.3390/cancers16173009 (PMC11394258; doi:10.3390/cancers16173009)
Supplement: Supplementary file 1 [file cancers-16-03009-s001.zip › Figure S1.pdf]

Figure S1

A

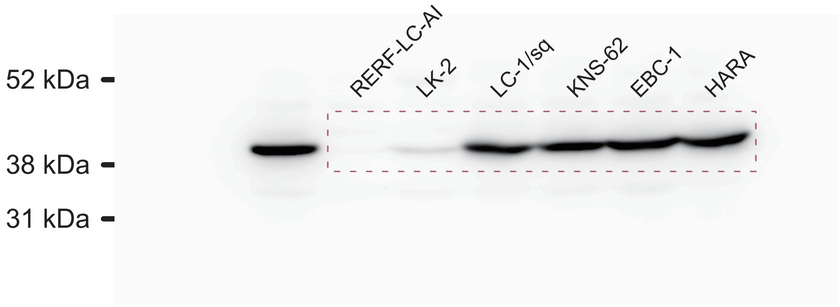

B

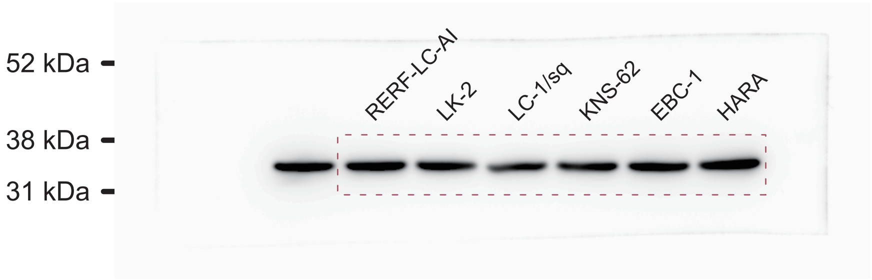

C

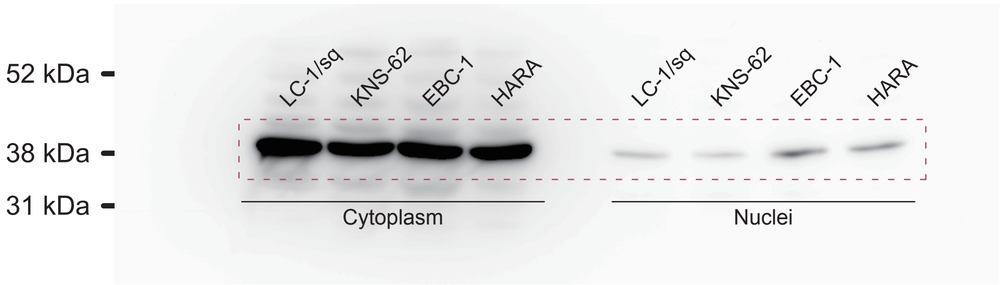

D

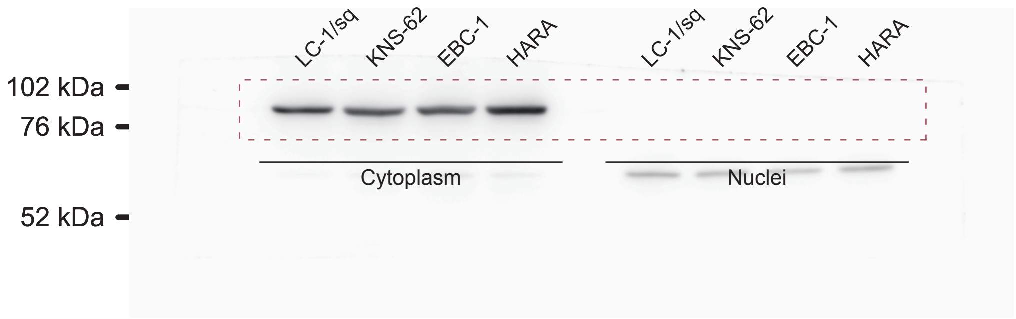

E

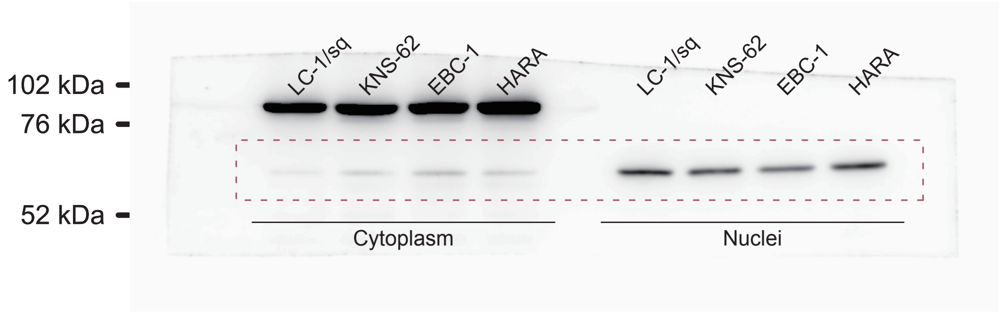

Figure S1 Whole western blot images in Figure 1. **(A, B)** Whole western blot images for maspin **(A)** and GAPDH **(B)** in the manuscript (Figure 1B). **(C, D, E)** Whole western blot images for maspin **(C)**, HSP90 **(D)**, and HDAC1 **(E)** in the manuscript (Figure 1D). The molecular weight of the sample was calculated using amersham full-range rainbow molecular weight marker and, sizes in kDa are indicated. Red dotted square indicates the proteins of interest.
