# Supplementary material for: Opposing Functions of Maspin Are Regulated by Its Subcellular Localization in Lung Squamous Cell Carcinoma Cells
Source: Cancers (Basel). 2024 Aug 29;16(17):3009. doi: 10.3390/cancers16173009 (PMC11394258; doi:10.3390/cancers16173009)
Supplement: Supplementary file 1 [file cancers-16-03009-s001.zip › Figure S2.pdf]

Figure S2

A

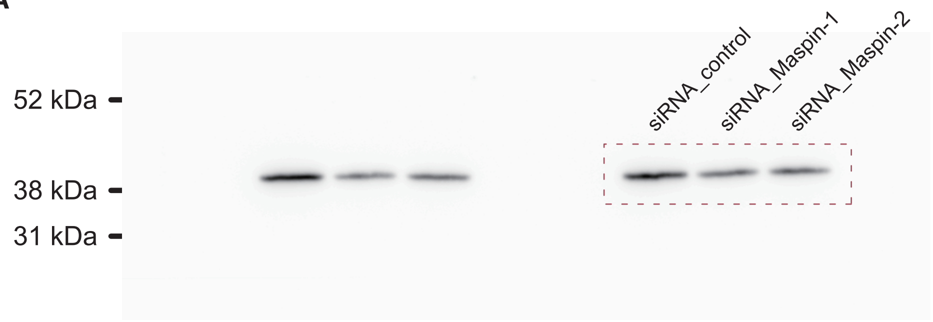

B

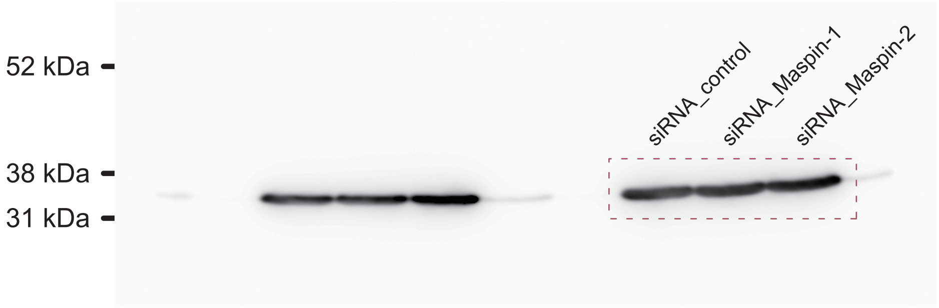

Figure S2 Whole western blot images in Figure 2. Whole western blot images for maspin **(A)** and GAPDH **(B)** in the manuscript (Figure 2A). The molecular weight of the sample was calculated using amersham full-range rainbow molecular weight marker and, sizes in kDa are indicated. Red dotted square indicates the proteins of interest.
