# Supplementary material for: Opposing Functions of Maspin Are Regulated by Its Subcellular Localization in Lung Squamous Cell Carcinoma Cells
Source: Cancers (Basel). 2024 Aug 29;16(17):3009. doi: 10.3390/cancers16173009 (PMC11394258; doi:10.3390/cancers16173009)
Supplement: Supplementary file 1 [file cancers-16-03009-s001.zip › Figure S3.pdf]

Figure. S3

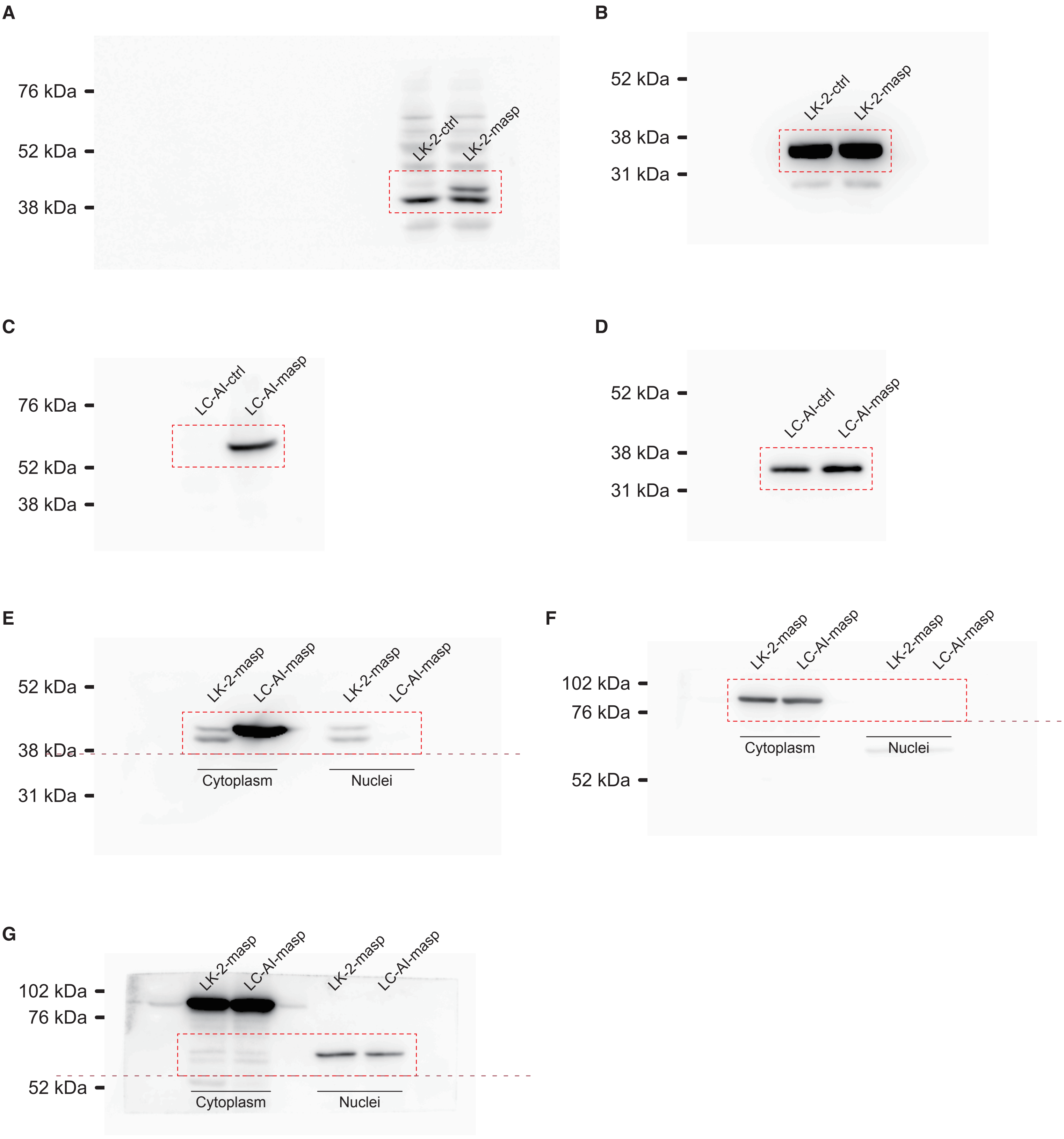

Figure S3 Whole western blot images in Figure 3. **(A, B, C, D)** Whole western blot images for maspin **(A, C)** and GAPDH **(B, D)** in LK-2 cells **(A, B)** and RERF-LC-AI cells **(C, D)** (Figure 3A). **(E, F, G)** Whole western blot images for maspin **(E)**, HSP90 **(F),** and HDAC1 **(G)** in the manuscript (Figure 3C). The molecular weight of the sample was calculated using amersham full-range rainbow molecular weight marker and, sizes in kDa are indicated. Red dotted square indicates the proteins of interest.
